# Deficient fear conditioning in psychopathy as a function of interpersonal and affective disturbances

**DOI:** 10.3389/fnhum.2013.00706

**Published:** 2013-10-25

**Authors:** Ralf Veit, Lilian Konicar, Jens G. Klinzing, Beatrix Barth, Özge Yilmaz, Niels Birbaumer

**Affiliations:** ^1^Institute of Medical Psychology and Behavioral Neurobiology, University of TübingenTübingen, Germany; ^2^International Centre for Ethics in the Sciences and Humanities, Research Training Group Bioethics, University of TübingenTübingen, Germany; ^3^Graduate School of Neural and Behavioural Sciences, International Max Planck Research School, University of TübingenTübingen, Germany; ^4^IRCCS Ospedale San CamilloVenezia-Lido, Italy

**Keywords:** fear conditioning, electrophysiology, skin conductance, psychopathy, emotional-cognitive interaction

## Abstract

The diminished fear reactivity is one of the most valid physiological findings in psychopathy research. In a fear conditioning paradigm, with faces as conditioned stimulus (CS) and electric shock as unconditioned stimulus (US), we investigated a sample of 14 high psychopathic violent offenders. Event related potentials, skin conductance responses (SCR) as well as subjective ratings of the CSs were collected. This study assessed to which extent the different facets of the psychopathy construct contribute to the fear conditioning deficits observed in psychopaths. Participants with high scores on the affective facet subscale of the Psychopathy Checklist-Revised (PCL-R) showed weaker conditioned fear responses and lower N100 amplitudes compared to low scorers. In contrast, high scorers on the affective facet rated the CS+ (paired) more negatively than low scorers regarding the CS− (unpaired). Regarding the P300, high scores on the interpersonal facet were associated with increased amplitudes to the CS+ compared to the CS−, while the opposed pattern was found for the antisocial facet. Both, the initial and terminal contingent negative variation indicated a divergent pattern: participants with pronounced interpersonal deficits, showed increased cortical negativity to the CS+ compared to the CS−, whereas a reversed CS+/CS− differentiation was found in offenders scoring high on the antisocial facet. The present study revealed that deficient fear conditioning in psychopathy was most pronounced in offenders with high scores on the affective facet. Event related potentials suggest that participants with distinct interpersonal deficits showed increased information processing, whereas the antisocial facet was linked to decreased attention and interest to the CS+. These data indicate that an approach to the facets of psychopathy can help to resolve ambiguous findings in psychopathy research and enables a more precise and useful description of this disorder.

## Introduction

Psychopathy is a personality disorder characterized by impaired affective processing and the persistent violation of the rights of others. The Psychopathy Checklist Revised (PCL-R) is a rating scale, widely used in research that gathers the multi faceted clinical construct of psychopathy. Factor analysis revealed four distinct, but intercorrelated facets (Hare, 2003). High scores on the first facet “interpersonal” describe a conning, manipulative, superficially charming person. The second facet captures “affective” deficits such as limited emotionality and lack of empathy. Both facets describe the core psychopathy feature already depicted by Cleckley (1941/1982) and covered by the original factor 1 proposed by Hare (1991). The other original factor 2 can also be subdivided into two facets: the “lifestyle” facet describes an impulsive, irresponsible and sensation seeking person, while the “antisocial behavior” facet encompasses mainly rule taking behavior and conflicts with the criminal law. There is an emerging consensus that factor 1 and 2 of the PCL-R represent two distinct components of psychopathy with different behavioral manifestations (Patrick and Bernat, 2009). It has been shown that both factors correlate in the opposed direction with measures of negative emotionality (Hicks and Patrick, 2006), supporting the multilayered construct of psychopathy. Moreover, different behavioral features of psychopathy may have a separate etiology (Patrick et al., 2007). For example, Molto et al. (2007) reported that response preservation deficits in psychopaths are linked to the antisocial feature but not to the interpersonal/affective characteristic of psychopathy. In the same vein, externalizing characteristics of psychopathy like antisocial behavior and substance abuse are said to be closely linked to factor 2 of the PCL-R (Patrick et al., 2005). Therefore, it is of special interest to reconsider well known psychopathy findings and their relation to subordinate PCL-R factors and related facets. Hereby it is possible to disentangle the etiological basis of certain psychopathy features.

One of the key findings in psychopathy is a reduced emotional responsiveness demonstrated by the diminished startle reflex potentiation during negative pictures (Lang et al., 1993; Patrick, 1994; Levenston et al., 2000; Vaidyanathan et al., 2009a). However, this finding depends strongly on the familiarity (Baskin-Sommers et al., 2013) and complexity (Sadeh and Verona, 2012) of the visual stimuli. Moreover, recent studies highlight the importance of attention modulation in instructed fear related tasks (Newman et al., 2010), which would undermine the thesis of a general impaired fear reactivity in psychopathy. Concerning the different components of psychopathy, the study of Patrick et al. (1993) revealed, that subjects scoring high on the affective component and low on antisocial behavior showed the strongest fear-potentiated startle deficit. Focusing on a continuous-compared to a discrete-analyses, a linear relationship between a reduced startle potentiation during aversive pictures and increasing scores in the interpersonal and affective facets of psychopathy was found (Sadeh and Verona, 2012).

Another important peripheral physiological finding in psychopathy research is the lack of electrodermal anticipatory fear responses to stimuli associated with punishment. This phenomenon was found in a countdown procedure (Hare et al., 1978; Ogloff and Wong, 1990), as well as in several delayed fear and aversive conditioning paradigms (Hare, 1965; Hare and Quinn, 1971; Flor et al., 2002; Veit et al., 2002; Birbaumer et al., 2005; Rothemund et al., 2012). Interestingly, psychopaths are able to recognize the relationship between unconditioned and conditioned stimuli on a cognitive level, but lack to decode the emotional importance of this association (Sommer et al., 2006).

Using functional neuroimaging, Birbaumer et al. (2005) showed that the physiological, as well as the cortical and subcortical fear deficits in psychopaths are related with factor 1 of the PCL-R and directly linked to amygdala, insular and orbitofrontal dysfunctions. The integrity of the amygdala is crucial for successful fear conditioning (Ledoux, 2000). Interestingly, structural imaging studies investigating the relationship between changes in brain morphometry and psychopathy subtypes could show that amygdala abnormalities (Yang et al., 2009) and gray matter reductions in the insula (De Oliveira-Souza et al., 2008) are strongly related to the affective and interpersonal facet of psychopathy.

While most of psychopathy research is based on peripheral psychophysiological measures, studies regarding event related potentials (ERP) are rarely published and show partly ambiguous results. Moreover only a few of those studies used the PCL-R as a diagnostic criterion for psychopathy (Kiehl et al., 1999, 2000, 2006; Flor et al., 2002; Howard and Mccullagh, 2007; Rothemund et al., 2012). Regarding the various ERP components, the N100 is sensitive to early attentional processes and larger amplitudes can be interpreted as a marker of selective attention (Luck et al., 2000). Jutai and Hare (1983) found in high -in comparison to low- psychopathic individuals decreased N100 amplitudes to task irrelevant stimuli indicating different allocation of attention depending on the focus of interest. Using an early event related potential (P140), Baskin-Sommers et al. (2012) similarly showed that psychopathic individuals are superior in focusing selectively on goal-directed information, whilst less attention is paid to peripheral, but fear and threat relevant information. Interestingly, the increase in N100 amplitude was associated with higher scores in the affective-interpersonal factor when emotional pictures were presented with high but not low complexity (Sadeh and Verona, 2012). Referring to fear conditioning paradigms the results are quite ambiguous: while Flor et al. (2002) found a larger N100 response to the paired stimuli (CS+) compared to the unpaired stimuli (CS−) during the acquisition phase in psychopaths, but not in healthy controls, Rothemund et al. (2012) reported smaller N100 components in psychopaths than controls, independent of the CS type. Another major electrical component is the P300 amplitude which is particular sensitive to changing salience of information (Sutton et al., 1965). Moreover this measure provides information of late attentional processes independent of early attentional allocations (Schupp et al., 2004). In general, the P300 potential is linked to orienting responses and reduced amplitude was found in many behavioral and medical disorders and diseases. For example, a reduction of the P300 amplitude was often reported as a diagnostic marker in schizophrenia (Galderisi et al., 2009). Reduced P300 amplitudes were shown in psychopaths compared to non-psychopathic participants in response to target stimuli in an oddball paradigm (Kiehl et al., 2000). In the context of Pavlovian aversive conditioning, the P300 was only occasionally addressed. During aversive conditioning, a CS+/− differentiation was specific for the psychopathic group during the early conditioning phase (Flor et al., 2002). Regarding the association with subordinate psychopathy factors, it has been shown the late positive potential that is similar to the P300 was negatively correlated with the affective-interpersonal dimension during the presentation of highly complex emotional pictures (Sadeh and Verona, 2012). This reinforces the assumption of a complex emotion-attention interaction which moderates emotional processing.

Another ERP component, which has been studied in psychopathy research, is the contingent negative variation (CNV). This slow changing cortical potential can be interpreted as a correlate of selective attention and arousal, but it is also sensitive to expectancy and motivational aspects (Tecce, 1972). The CNV occurs as a response to a two-stimulus paradigm which consists of a warning signal indicating the condition followed by an imperative signal. The resulting potential shift can be decomposed into an initial (iCNV) and a terminal (tCNV) component. While the initial orienting response to the warning stimulus is associated with evaluation of the stimulus (Rockstroh et al., 1982), the terminal CNV arises just before the onset of the second stimulus and is modulated by its emotional salience and particularly pronounced in anticipation of intense aversive stimuli, i.e. an electric shock (Birbaumer et al., 1990). Initially, it was reported that psychopaths display a diminished CNV to the warning stimulus (McCalloum, 1973). Later findings indicated that psychopaths showed even enhanced CNV responses when the task is sufficiently interesting (Jutai and Hare, 1983). Forth and Hare (1989) used a forewarned reaction time task in which the participants could win or lose money. They found an enhanced magnitude of the iCNV, but not the tCNV in psychopaths, compared to non-psychopaths. Studies exclusively focusing on electrophysiological correlates of fear conditioning are generally rare (e.g., Lumsden et al., 1986; Regan and Howard, 1995). In healthy participants, Regan and Howard (1995) reported increased tCNV amplitudes to paired stimuli (CS+) compared to unpaired stimuli (CS−) in anticipation of phobia-related animal pictures. Regarding psychopathic individuals, Flor et al. (2002) used foul odor as the (US) and emotionally neutral faces as conditioned stimuli. They found superior information processing in psychopaths indicated by increased tCNV independent of CS type. Recently, Rothemund et al. (2012) used a fear conditioning paradigm with electric shocks as the US and reported larger left lateralized CS+/CS− differentiation in the iCNV during the late acquisition phase in psychopaths. The terminal component revealed a mixed pattern with lower magnitudes in psychopaths compared to non-psychopaths at frontal sites and the reversed pattern at parietal sites. Nevertheless it is important to mention that it is still unclear, if the inconsistent results in the ERP findings are due to the different tasks, stimuli or subjects. Despite the strong evidence of deficient fear responses that were found in several experimental investigations in general psychopathy, to date, only little work has been done to investigate the relationship between the subtypes of psychopathy in relation to their possible psychophysiological correlates. Both Flor et al. (2002) and Rothemund et al. (2012) did not explicitly differentiate between lower psychopathy factors and assumed a general factor that contributes to the fear conditioning deficit. The study of Birbaumer et al. (2005) investigated only subjects with high values on factor 1 and led to the conclusion that factor 1 is the mediating factor, influencing fear reactivity during implicit learning. Despite the growing evidence of affective-interpersonal characteristics that modulate fear related learning (Patrick, 1994), there is only limited information on the subcomponents of psychopathy specifically regarding the facets lifestyle and antisociality. Furthermore, disentangling the different facets of psychopathy would, on one hand be fruitful to extend various theoretical models (and related focuses as well as etiologies) and on the other hand may shed more light on prognosis (Marcus et al., 2006).

The present study aimed to expand the limited knowledge of Pavlovian fear conditioning on the subcomponents of psychopathy in highly criminal psychopaths. Therefore, we wanted to assess to which extent the different factors and facets of Hare's psychopathy construct contribute to the conditioned fear deficit on the peripheral, subjective and electrocortical level. In subjects with increasing PCL-R scores in the affective and interpersonal facet we expected a diminished anticipatory skin conductance response to the CS+ compared to CS− during the acquisition phases in the classical conditioning task. Furthermore we wanted to investigate differences in the subjective and electrodermal fear responses at the facet level in more detail.

Another focus of the study were the electrocortical correlates of fear conditioning in relation to the subordinate psychopathy dimensions. We hypothesized in an exploratory manner, that the higher the subjects score on the antisocial facet of the PCL-R, the better they are able to differentiate between CS+ and CS− regarding the measured P300 and CNV components. Contrary, we expected higher scores on the affective and/or interpersonal facet to be associated with a deficit in CS+/CS− differentiation. Concerning the N100 component we hypothesized, that subjects with high scores on the affective or interpersonal facet will show more pronounced amplitudes to the CS+, than to the CS−.

## Materials and methods

### Participants

Fourteen adult psychopathic males (mean age: 43.14 ± 11.52 years, all right handed) with a history of violent and/or sexual offences participated in the study. All of them were long-time detained in one of two cooperating German maximum security forensic psychiatric institutions. Exclusion criteria were an age below 18 or over 55 years, an IQ below 80 or health problems. The participants were informed about the aim of the study and gave written informed consent. They were paid 20€ in agreement with the forensic institutions. All subjects were scored using Hare's Psychopathy Checklist Revised (PCL-R) (Hare, 2003), resulting in a mean score of 30.14 ± 2.77 (range = 25–34). Nine out of fourteen participants reached the cut-off score of 30, which is commonly used in American studies to classify psychopaths. However, all participants exceeded the psychopathy score of German and European norms (Cooke et al., 2004). Regarding the two main factors of the PCL-R, the mean scores were 11.79 ± 2.51 for factor 1 and 15.86 ± 1.56 for factor 2. According to the four facet model the mean scores were: Interpersonal facet: 5.50 ± 1.65; affective facet: 6.29 ± 1.35; lifestyle facet 7.00 ± 1.35; antisocial facet 15.86 ± 1.56. All participants met the full criteria for a diagnosis of antisocial personality disorders in line with the DSM-IV criteria (Apa, 2000). The study was approved by the Ethics Committee of the Medical Faculty of the University of Tübingen. The classical conditioning experiment presented here was conducted in the context of a comprehensive study on criminal psychopaths.

### Experimental design

A classical delayed fear conditioning paradigm was applied, using a modified version of the design of Birbaumer et al. (2005). Two neutral grey-scale male faces were used as conditioned stimuli (Schneider et al., 1994). With pseudo-random assignment between participants, one of the faces was paired with the unconditioned stimulus (CS+), while the other face was never followed by the unconditioned stimulus (CS−). The (US) consisted of an electric shock and was administered using a Digitimer DS5 Isolated Bipolar Constant Current Stimulator (Digitimer Ltd., United Kingdom). The two electrodes were attached over the distal and proximal phalanx of the right thumb. The intensity of the electric shock was individually adjusted in a pre-experiment, immediately before the actual experiment, at a level where the participants estimated the electric shock as unpleasant (“8” on a visual analog scale in which “0” indicates not at all unpleasant and “10”, extremely unpleasant). The faces were presented for 5 s and the US was administered after 4 s of the picture presentation and lasted 500 ms.

A 50% partial reinforcement schedule was chosen, indicating that only 50% of the CS+ were followed by US. The conditioning procedure consisted of a habituation phase (6 CS+, 6 CS−, 4 US), an acquisition phase (32 CS+, 32 CS−) separated in an early and late acquisition block, and finally an extinction phase (16 CS+, 16 CS−). During habituation and extinction, the Inter-Trial-Interval (ITI) varied between 4.5 and 7.5 s, during acquisition between 7.5 and 12 s.

Subjective ratings of emotional valence and arousal were collected on a 9-point scale using the Self Assessment Manekin (Bradley and Lang, 1994) four times (after habituation, early acquisition, late acquisition and extinction). Based on the inherent implicit learning mechanism in classical conditioning it was not intended to inform the subjects about the CS-UCS contingency. The only information that was provided was that male faces will be shown and electric shocks will be applied from time to time. However, the contingency of US and CS was assessed after both acquisition phases and after the extinction phase using a visual analogue scale (“How likely is it that an electric stimulus will follow this face?” (ranging from “1,” indicating no association between CS and US, to “9,” indicating an absolute certainty that US follows CS).

### Apparatus and physiological recordings

Physiological data were acquired using a Theraprax Neurofeedbacksystem (NeuroConn GmbH, Ilmenau, Germany). Electroencephalography (EEG), electrooculography (EOG), and skin conductance response (SCR) were recorded using a sampling rate of 128 Hz and 40 Hz high cut-off-filtering. EEG activity was measured using four recording electrodes, located at Fz, FCz, Cz, and Pz according to the 10–20 system. Left mastoid was used as the reference and right mastoid as ground. SCR activity was recorded from the intermediate phalanx of the index and ring finger of the non-dominant hand with strap electrodes.

### Data processing

Skin conductance data were analyzed using the Matlab-based software Ledalab 3.4 (Benedek and Kaernbach, 2010). The recordings were decomposed into their tonic and phasic components which resulted in phasic activity timelines with zero baselines. SCR was then extracted for each single trial using the integrated phasic SCR between 1 and 4 s after picture onset and averaged over paired and unpaired trials in the different conditioning phases. This novel approach allows an unbiased estimation of sympathetic fear-related activity compared to the conventional baseline to peak computation using minimum amplitude criteria for classifying SCR.

The EEG data were processed using BrainVision Analyzer Professional 2.01 (BrainProducts GmbH, Gilching, Germany). A notch filter at 50 Hz was applied. Thereafter the signals were filtered using a 0.1 Hz highpass and a 15 Hz lowpass filter. Ocular artifacts were adjusted using an eye-blink artifact correction method (Gratton et al., 1983). The data was then segmented in epochs of 5.5 s duration (−0.5 to 5 s relative to the onset of the CS). On the segmented data, a time interval of 300 ms was used to detect and reject remaining artifacts exhibiting (a) gradient changes more than 15 μV/ms, (b) voltage differences of more than 100 μV, or c) signal amplitudes over ±80 μV. Baseline correction was performed using a 200 ms interval before trial onset. ERPs were extracted using the following parameters: For N100, amplitude and latency of the highest voltage peak between 100 and 220 ms was registered, while 300–800 ms was used for P300. CNV areas under the curve were calculated for the time intervals 800–1500 ms (iCNV) as well as 3500–4000 ms (tCNV).

### Statistical analysis

The self-report and SCR data were analyzed by means of repeated-measures analyses of variance (ANOVA) with phase (habituation, acquisition and extinction) and CS-type (CS+ vs. CS−) as within-group factors. Contingency ratings were analyzed using the acquisition phase (early vs. late) and the CS-type as within subject factor. For each EEG parameter separate repeated measures ANOVAs were performed using electrode position (FCz, Fz, Cz, and Pz), and CS-type as within-subject factors during habituation and extinction. During acquisition, the first and second half of the phase (early and late acquisition block) were selected as an additional within-subject factor. Greenhouse-Geisser correction was applied if the sphericity assumption was violated. In order to improve signal-to-noise ratio in subsequent analysis, EEG amplitudes during early and late acquisition phase were averaged over corresponding CS type. Differences between the CS types during early and late acquisition as well as both acquisition blocks combined were calculated. The differential values were correlated with the individual total PCL-R score as well as with its two factors and underlying facets (Pearson's bivariate correlation). Apart from simple correlations a partial correlation approach was conducted using one facet as independent variable and the respective other facets as control variables. In addition, a forward stepwise regression analysis using the four facets as independent variables and the differential (CS+ vs. CS−) subjective, peripheral and EEG parameters as dependent variables. For the inclusion we used F-probability of 0.05 and for the exclusion a probability of 0.10. Furthermore, a multiple regression analysis with all four facets at once was computed to determine the account of variance. Because of problems during SCR recordings, data were available only for 11 participants. One participant was excluded from the EEG analyses due to artifacts during data acquisition.

## Results

### Correlation between subjective, electrodermal and EEG measures

The correlation analyses revealed a close relationship between the differential SCR responses and N100 and P300 amplitudes during early and late acquisition. Reduced anticipatory fear responses were accompanied with degraded early attention to the CS+ compared to the CS−, as reflected in the N100 waveforms. Late attentional processes, as reflected in the P300 potential showed the opposite effect. In a similar vein, a smaller conditioned SCR was correlated with a more negative rating of CS+ faces (see Table 1).

**Table 1:** **Correlation between measures**.

	**SCR**	**Valence**	**N100**	**P300**	**iCNV**	**tCNV**
**CORRELATION BETWEEN MEASURES EARLY ACQUISITION PHASE**						
SCR	1					
Valence	0.53	1				
N100	−0.63^*^	−0.34	1			
P300	−0.55	−0.31	0.69^*^	1		
iCNV	−0.12	0.08	−0.54	0.03	1	
tCNV	−0.47	−0.22	−0.16	0.25	0.82^**^	1
**CORRELATION BETWEEN MEASURES LATE ACQUISITION PHASE**						
SCR	1					
Valence	0.41	1				
N100	−0.65^*^	0.08	1			
P300	−0.65^*^	−0.26	0.45	1		
iCNV	−0.17	−0.39	0.03	0.08	1	
tCNV	0.14	0.04	−0.16	0.05	0.40	1

* p < 0.05,

** p < 0.01. The correlations refers to the difference between CS+ and CS− of the respective variables.

### Subjective and skin conductance measures

**Figure 1:**
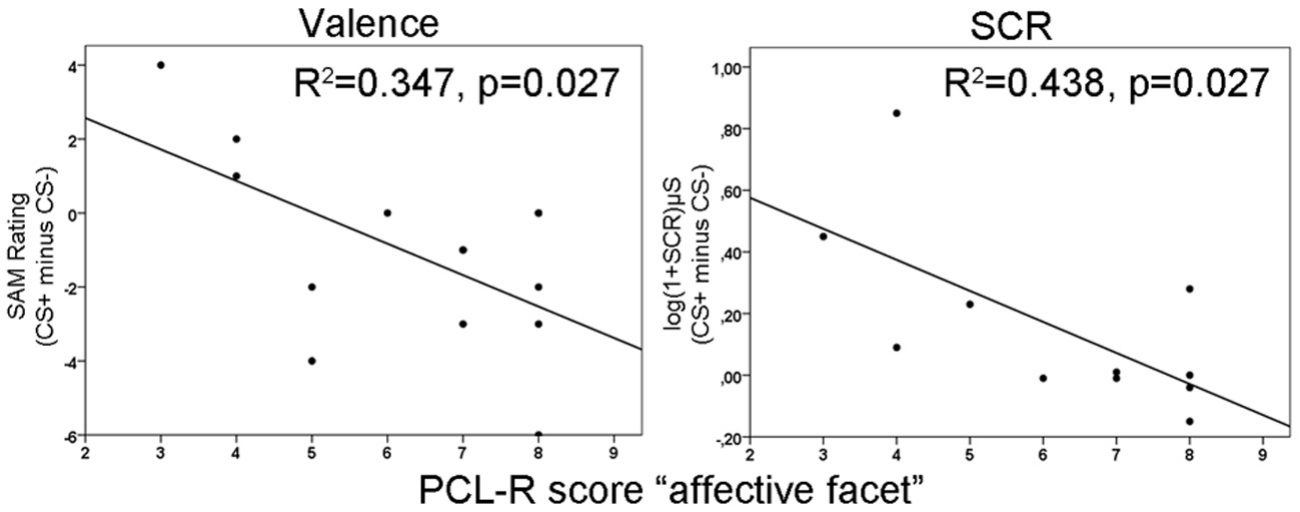
**Illustration of negative correlations between differences in valence ratings (left) and skin conductance responses (right) with the affective facet of the Psychopathy Checklist Revised (PCL-R) during the early acquisition phase.** *R*^2^-values are depicted for the significant predictors in a stepwise regression analysis.

Repeated measurement ANOVAs with the factors phase (habituation, early acquisition, late acquisition and extinction) and CS type revealed no significant main effects or interactions for valence and arousal as well as for SCRs during fear conditioning. However, a significant difference in contingency ratings between CS types [*F*_(1, 13)_ = 45.66, *p* < 0.0001] was found indicating, that processing of the paired and unpaired stimuli was intact on a cognitive level. The correlation analysis revealed that during early acquisition high total PCL-R scores were negatively associated with differential SCR amplitudes (*r* =−0.757, *p* = 0.007). Regarding the different facets, the effect was most prominently induced by the affective facet of psychopathy (*r* = −0.663, *p* = 0.036; see Figure 1, right). Partial correlation analysis using the affective facet as dependent variable and the other facets as control variable confirmed the observed association, although it became not significant due to the reduced degrees of freedom relative to the number of control variables (*r* = −0.604, *p* = 0.112). A stepwise regression analysis revealed that the reduced differential SCR responses during the early acquisition phase were solely predicted by the affective facet [*R*^2^ = 0.439, [*F*_(1, 9)_ = 7.04, *p* = 0.026]. The other facets did not explain additional variance. Concerning the differential valence ratings, we found the opposite effect. Participants scoring high on the affective facet rated the paired stimulus more negative than the unpaired stimulus in the early acquisition phase (*r* = −0.589, *p* = 0.027; see Figure 1 **left**). This effect was confirmed using partial correlation analysis (*r* = 0.608, *p* = 0.050). During late acquisition phase, no significant correlations between subjective measures, SCR and PCL-R scores were found. Contingency ratings revealed that the CS-UCS pairing was correctly identified during the early [*t*_(13)_ = 4.63, *p* < 0.001] and late [*t*_(13)_ = 7.43, *p* < 0.001] acquisition phase. All zero-order correlations and partial correlations for the subjective and SCR measures are depicted in Table 2.

**Table 2:** **Correlation between PCL-R scores and peripheral and subjective measures**.

**Acqusition CS+ - CS−**	**PCL-R total**	**Factor 1**	**Factor 2**	**Facet 1**	**Facet 2**	**Facet 3**	**Facet 4**	***R*^2^**
SCR early	−0.76^**^	−0.62	−0.28	−0.66^*^	−0.21	−0.53	0.35	0.61
				−0.60				
SCR late	−0.28	0.21	0.07	0.16	0.15	0.43	−0.58	0.62
SCR all	−0.34	−0.31	−0.15	−0.39	−0.03	−0.02	−0.25	0.38
Valence early	−0.32	−0.17	−0.21	−0.59^*^	0.37	−0.34	0.11	0.60
				−0.61^*^				
Valence late	0.04	0.19	−0.26	−0.07	0.37	−0.13	−0.22	0.17
Valence all	−0.12	0.06	−0.29	−0.33	0.44	−0.25	−0.11	0.36

*Each cell consists of simple correlation coefficients. Partial correlations using all facets as control variable are listed as the second value in the cells. R^2^ including all facets*.

* *p < 0.05*,

** *p < 0.01*.

### Cortical measures (EEG)

#### N100

Repeated measures ANOVA revealed no main effect or interaction for the N100 amplitude during the habituation and acquisition phase. During extinction, a significant electrode effect was observed [*F*_(3, 36)_ = 4.172, *p* = 0.034] with larger amplitudes at Fz compared to Pz. The correlation analyses showed a significant positive covariation between the scores in the affective facet of the PCL-R and the differential N100 amplitude (CS+ minus CS−) at frontal locations (FCz *r* = 0.588, *p* = 0.035; Fz *r* = 0.585, *p* = 0.036) during the early acquisition phase, indicating decreased attentional allocation to the CS+ in comparison to the CS−. Correlation analyses using both acquisition phases combined revealed a tendency of decreased N100 amplitudes during CS+ compared to CS− with the interpersonal facet (*r* = 0.524, *p* = 0.066). A partial correlation analyses confirmed this highly significant association (*r* = 0.903, *p* < 0.001). During extinction, a negative correlation was observed between total PCL-R score and CS+ /CS− differentiation (*r* = −0.565, *p* = 0.044).

**Figure 2:**
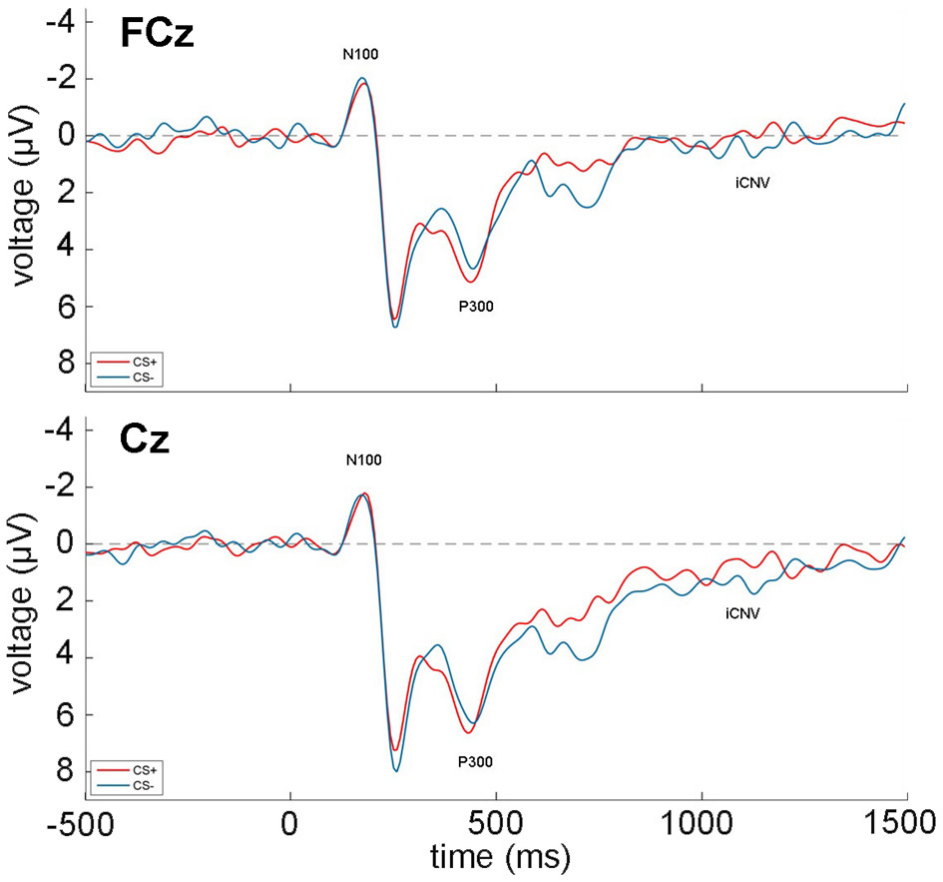
**Grand average of the EEG recordings (N100, P300, iCNV) over all participants and acquisition trials at FCz (top) and Cz (bottom).** CS+ trials are depicted in red, while CS− trials are shown in blue. Time 0 indicates the onset of the face stimuli.

The grand averages during acquisition of the EEG recordings at FCz and Fz over all participants and trials are shown in Figure 2. All zero-order correlations and partial correlations between psychopathy scores and EEG measures are depicted in Table 3.

**Table 3:** **Correlation between PCL-R scores and EEG measures**.

**Acqusition CS+ - CS−**	**PCL-R total**	**Factor 1**	**Factor 2**	**Facet 1**	**Facet 2**	**Facet 3**	**Facet 4**	***R*^2^**
N100 early	0.44	0.49	−0.11	0.59^*^	0.09	0.19	−0.39	0.39
				0.54				
N100 late	−0.07	0.03	−0.06	−0.12	0.20	−0.32	0.32	0.41
N100	0.13	0.33	−0.25	0.00	0.52	−0.39	0.14	0.85
					0.90^**^			
P300 early	0.58^*^	0.53	0.37	0.68^*^	0.04	0.46	−0.08	0.53
				0.63^*^				
P300 late	−0.70^**^	−0.69^**^	−0.18	−0.42	−0.58^*^	−0.65^*^	0.55^*^	0.49
						−0.52		
P300	−0.23	−0.47	0.47	−0.05	−0.67^*^	−0.00	0.74^**^	0.79
							0.77^**^	
iCNV early	0.14	−0.12	0.75^**^	−0.09	−0.08	0.38	0.52	0.63
iCNV late	−0.09	−0.28	0.41	0.00	−0.43	0.11	0.42	0.33
iCNV	−0.18	−0.45	0.58^*^	−0.12	−0.56^*^	0.04	0.73^**^	0.69
							0.70^*^	
tCNV early	0.31	0.16	0.67^*^	0.32	−0.12	0.51	0.27	0.51
tCNV late	−0.07	−0.33	0.18	0.06	−0.58^*^	−0.17	0.45	0.39
tCNV	−0.00	−0.25	0.40	0.18	−0.61^*^	−0.05	0.60^*^	0.59
					−0.47		0.54	

*Each cell consists of simple correlation coefficients. Partial correlations using all facets as control variable are listed as the second value in the cells. R^2^ including all facets*.

* *p < 0.05*,

** *p < 0.01*.

#### P300

No significant differences were found during the habituation phase. During acquisition significantly increased P300 amplitudes at parietal compared to frontal sites were observed [*F*_(3, 36)_ = 8.18, *p* = 0.001]. In addition, a significant electrode × block interaction was found [*F*_(3, 36)_ = 4.95, *p* = 0.023]. During the extinction phase, a significant electrode effect [*F*_(3, 36)_ = 4.351, *p* = 0.010] with larger P300 amplitudes at parietal compared to frontal sites was found. The correlation analysis revealed that high PCL-R scorers had an augmented P300 amplitude to the CS+ compared to the CS− in the early condition phase at all recording sites, but most prominent at the parietal electrode (FCz: *r* = 0.575, *p* = 0.040; Fz: *r* = 0.599, *p* = 0.031; Cz: *r* = 0.560, *p* = 0.047; Pz: *r* = 0.816, *p* = 0.001). The opposite pattern was found in the late acquisition phase with decreased P300 amplitude to CS+ in relation to the CS− at fronto-central positions (FCz: *r* = −0.703, *p* = 0.007; Fz: *r* = −0.674, *p* = 0.011, Cz: *r* = −0.702, *p* = 0.007). The correlation analysis, using the combined early and late conditioning phase, revealed a positive association between the P300 amplitude differentiation (CS+ minus CS−) and the antisocial facet of the PCL-R (Fz: *r* = 0.801, *p* = 0.001; Cz: *r* = 0.736, *p* = 0.004). The interpersonal facet of the PCL-R covaried negatively with the P300 differentiation (Cz: *r* = −0.671, *p* = 0.010; see Figure 2). Partial correlation analysis showed that the antisocial facet was the strongest predictor (*r* = 0.774, *p* = 0.009) for the CS+/CS− differentiation in the P300 amplitude. In a similar vain, the stepwise regression analysis favored a model with antisocial facet as the solely predictor [*R*^2^ = 0.541, *F*_(1, 12)_ = 12.98, *p* = 0.004]. During extinction no correlation reached statistical significance.

### Initial contingent negative variation (iCNV)

During habituation and extinction, no significant effects were found. During acquisition, a significant electrode effect [*F*_(3, 36)_ = 6.545, *p* = 0.004] with increased negativity at fronto-central electrode position was observed. However, neither CS type nor acquisition blocks yielded statistically significant effects. The correlation analyses revealed a positive relationship between the CS type related iCNV differentiation (CS+ minus CS−) with the antisocial facet (Fz: *r* = 0.733, *p* = 0.004; FCz: *r* = 0.716, *p* = 0.006; Cz: *r* = 0.623, *p* = 0.023) as well as with the original factor 2 of the PCL-R (Fz: *r* = 0.583), and in particular during the early acquisition phase (Fz: *r* = 0.747, *p* = 0.003). High scores on the antisocial facet were associated with smaller negative and even positive shifts of brain activity in response to CS+ compared to the CS−. Moreover, the interpersonal facet of the PCL-R covaried negatively with the CS+/CS− differentiation (Fz: *r* = −0.561, *p* = 0.036; Pz: *r* = 0.602, *p* = 0.029). Thus, participants with high interpersonal deficiencies had larger negative shifts in their brain activity in response to paired (CS+) relative to unpaired (CS−) stimuli (see Figure 4). A partial correlation analysis revealed that the antisocial facet showed the strongest association (*r* = 0.698), just as the stepwise regression analysis yielded [*R*^2^ = 0.537; *F*_(1, 12)_ = 12.77, *p* = 0.004]. During extinction, a negative correlation between scores in the antisocial facet and CS+/CS− iCNV differentiation was observed (*r* = −0.588, *p* = 0.035).

**Figure 3:**
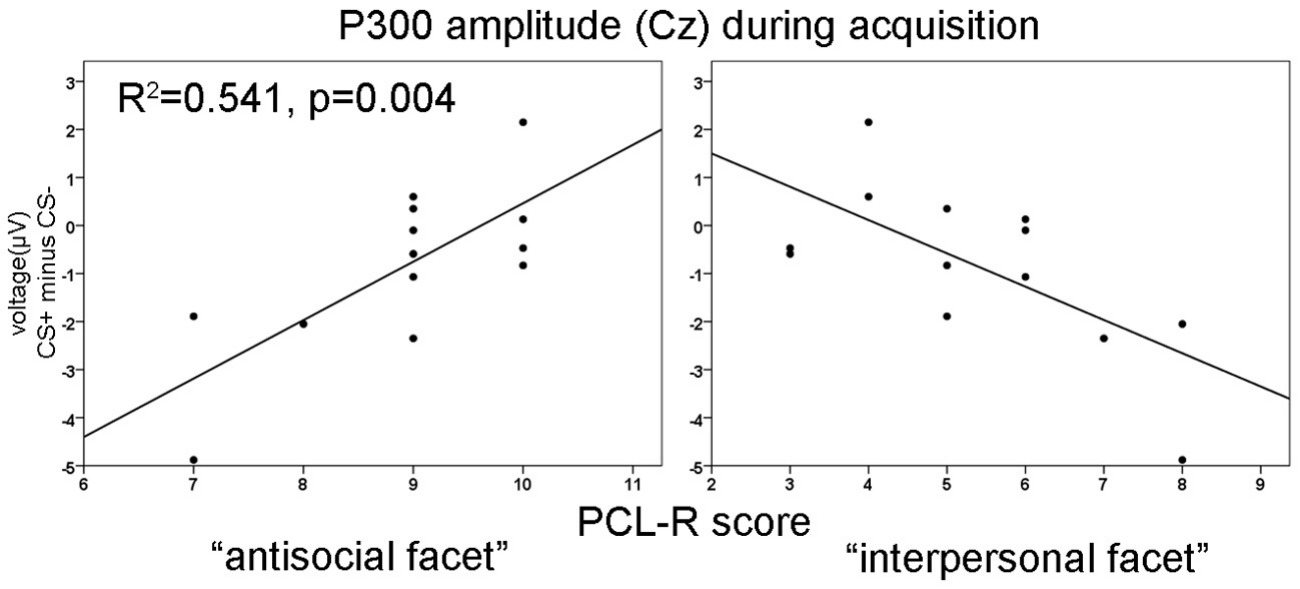
**Correlation of P300 amplitude differences at Cz with the antisocial (left) and the interpersonal facet (right) of the PCL-R during acquisition phase.** *R*^2^-values are depicted only for the most significant predictor in a stepwise regression analysis.

**Figure 4:**
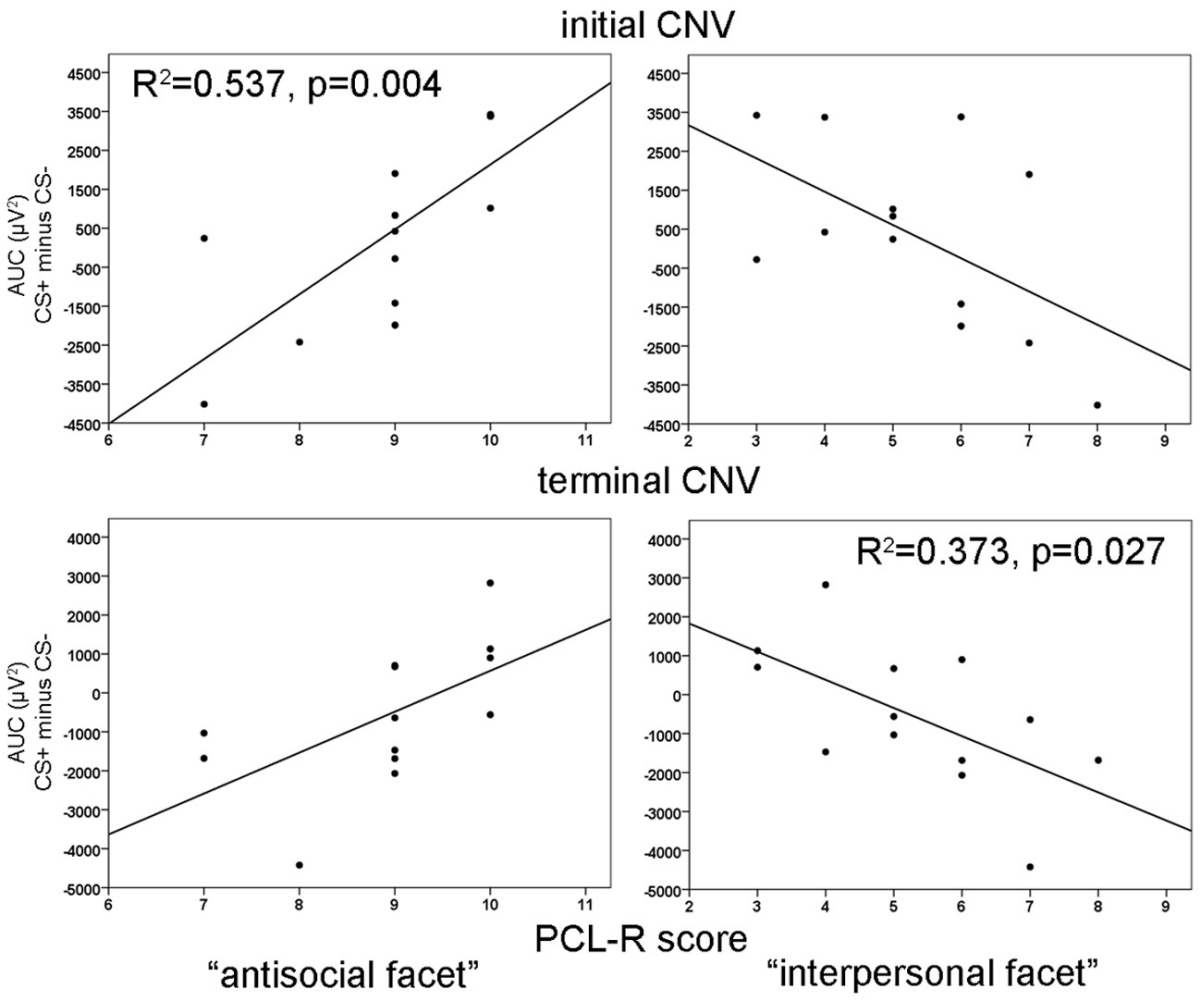
**Correlation of iCNV (top) at Fz and tCNV (bottom) at Cz area-under-the-curve (AUC) differences with the antisocial facet (left) and the interpersonal facet (right).** *R*^2^-values are depicted only for the most significant predictor in a stepwise regression analysis.

### Terminal contingent variation (tCNV)

ANOVA revealed no significant effects during habituation or extinction. During the acquisition phase, there was only a tendency of an electrode effect [*F*_(3, 36)_ = 2.64, *p* = 0.064] with pronounced tCNV at Cz and the smallest effect at Pz, but no CS type or blocks effect. Resembling the iCNV results, the correlation analyses showed that the CS+/CS− differentiation in the terminal CNV was raised by the antisocial and interpersonal psychopathy facet. While high scores of the antisocial facet correlated positively with CS+/CS− tCNV differentation at fronto-central sites (Fz: *r* = 0.645, *p* = 0.017; FCz: *r* = 0.654, *p* = 0.015, Cz: *r* = 0.595, *p* = 0.032), the interpersonal facet revealed a negative correlation centrally (Cz: *r* = 0.610, *p* = 0.027). Thus, high antisocial scores were again associated with smaller negative or even positive shifts in brain activity in CS+ trials, while an opposite pattern was found in participants with pronounced interpersonal deficits. A step-wise regression analysis showed that a model including the interpersonal facet only, explained most variance [*R*^2^ = 0.373, *F*_(1, 12)_ = 6.53, *p* = 0.027]. During extinction, a negative correlation between scores in the lifestyle facet and the tCNV difference between CS+ and CS− was observed (*r* = −0.566, *p* = 0.044).

### Discussion

The present study aimed to explore associations of psychopathy and the different facets in Hare's psychopathy construct with subjective, peripheral-physiological and cortical measurements in a classical fear conditioning paradigm. On the group level, without accounting for the individual PCL-R scores, we did not find a conditioning effect in the subjective and peripheral measures in these highly psychopathic criminals. The results of our study corroborate previous findings, demonstrating a psychopathy related deficiency in developing a conditioned fear response to aversive or fearful stimuli (Flor et al., 2002; Veit et al., 2002; Birbaumer et al., 2005; Rothemund et al., 2012). In addition, the correlation analyses indicated that the deficiency in fear conditioning is linearly modulated by total PCL-R scores. Participants with extremely high psychopathy scores showed weaker or absent conditioned electrodermal fear responses compared to lower scorers. A closer inspection revealed that this effect was most prominently modulated by the affective facet. The opposed pattern was found using the differences in valence ratings between CS+ and CS− as a subjective measure of successful conditioning. Interestingly, the correlation between the differential valence ratings and SCR responses revealed that subjects with profound fear deficit rated the CS+ faces more negative than the CS−. We postulate that participants scoring high on the affective facet either tried to mimic normal emotional behavior by responding more negatively to the paired stimuli or that they are indeed perfectly able to evaluate the expected “cognitive” dimension of fear. This matches the observation that core psychopaths are masters of deception and/or are cognitively quite aware of the contingency between face stimuli and painful electric shock. Recently, Lopez et al. (2013) assessed self-reported psychopathy (PPI-R, The Psychopathic Inventory Revised), (Lilienfeld and Widows, 2005) in a student sample and showed that high scores in the “fearless dominance” subscale, but not in the “impulsive antisociality” subscale, were associated with deficient fear conditioning. This is similar to what we found in highly criminal psychopaths. The diminished emotional responsiveness is a key finding in psychopathy research and it has been shown that the reduced startle response during presentation of negative emotions is closely related to factor 1 of the PCL-R (Patrick et al., 1993; Patrick, 1994; Vaidyanathan et al., 2009b). Those findings fit perfectly to the elaborated theoretical framework of Patrick et al. (2007), which describes a bifactor conceptualization of psychopathic syndromes with different underlying etiological mechanisms. Nonetheless, it is not yet clear whether the affective or the interpersonal facet contributes to the fear deficit or to which extent they might contribute. Based on the anticipatory skin conductance responses, our data indicate that in a fear conditioning paradigm, the affective facet modulates the deficit in fear reactivity.

Regarding the EEG measures, we found decreased N100 amplitudes to the CS+ compared to the CS− during the early acquisition phase in participants with pronounced affective deficits. The same association was found in participants with distinct interpersonal deficits, when regarding the early and late acquisition phase combined. This result might reflect a different early attention status, presumably arising from the conditioning procedure in psychopaths scoring high on the superordinate factor 1 of the PCL-R. Our findings are in contrary to Flor et al. (2002) who revealed increased N100 amplitudes in the psychopaths to CS+ compared to CS− trials in the early conditioning phase, but in line with Rothemund et al. (2012) who showed overall lower N100 amplitudes in psychopaths compared to healthy controls. In context of the response modulation theory, Newman et al. (2010) proposed attentional abnormalities in psychopathy as an alternative fear deficit explanation. Furthermore, in a recent study deficient fear responses in highly psychopathic individuals were only found when the attention was shifted to irrelevant information at early stages prior to the onset of fear-relevant stimuli (Baskin-Sommers et al., 2011). We could show that the phase specific CS type differentiation in the N100 amplitude was directly related to the differential SCR responses in the early and late acquisition phase. Higher N100 amplitudes to the CS+ compared to the CS− were accompanied with enhanced electrodermal reactivity to the CS+, supporting the importance of early sensory processing of conditioned stimuli in fear conditioning (Miskovic and Keil, 2012). Although a direct comparison between our findings and the results of Newman et al. (2010) and Baskin-Sommers et al. (2011) is not possible, due to critical differences ranging from the experimental paradigm (implicit vs. explicit learning) up to the data collection (anticipatory SCR's and ERP's vs. fear potentiated startle response), our findings highlight the influence of early attentional processes in fear related learning that might be different in participants with affective/interpersonal deficits.

Regarding the P300 component, correlational analyses revealed larger CS type differentiation in the P300 amplitudes in high PCL-R scorers during the early acquisition phase. Considering the late acquisition phase, however a negative correlation was found with the PCL-R total scores as well as with factor 1 and the P300 responses. Flor et al. (2002) found a comparable positive CS+/− differentiation at frontal leads only in the psychopathic group during the early acquisition period, while Rothemund et al. (2012) showed a CS type differentiation in both psychopathic and non-psychopathic individuals. Our findings are consistent with the view that high psychopathic individuals exhibit intact attentional processes in particular during the early conditioning phase, when the meaning of the situation must be conceived. With respect to the psychopathy facets we showed that the interpersonal facet was negatively correlated with the CS+/− evoked P300 responses throughout the acquisition, while the antisocial facet modulated the P300 responses in the opposite way. It has to be emphasized that the P300 does not reveal a uniform pattern in psychopathy and most studies reporting reduced P300 amplitudes in psychopathy (Kiehl et al., 1999, 2000, 2006; Gao and Raine, 2009) used paradigms that influence P300 pattern selectively. In a similar vein, Patrick et al. (2006) reported a strong association between the reduction in P300 amplitude and externalizing dimensions such as antisocial behavior, pathological gambling, drug abuse and disinhibition in a visual oddball paradigm. Accordingly, this approach explains the decreased ERPs in psychopaths, scoring high on the interpersonal but not on the antisocial facet. However, it is important to mention that the P300 potential is not a measure for successful or unsuccessful Pavlovian fear conditioning, instead it measures selective attention and expectancy, modulating emotional learning (Verleger et al., 1994). Regardless of psychopathy scores, we demonstrated that enhanced P300 to the conditioned stimuli was associated with diminished electrodermal fear responses, suggesting cognitive top-down modulation in affective learning (Olofsson et al., 2008).

Concerning the conditioned CNV responses related to expectancy, orientation (iCNV) and preparation (tCNV), we found increased cortical negativity in the CS+ compared to the CS− condition in frontal and central sites for participants scoring high on the interpersonal facet. On the contrary, high scorers on the antisocial facet showed increased negativity in CS− compared to CS+ trials. Augmented iCNV was observed in some studies investigating psychopaths (Forth and Hare, 1989; Flor et al., 2002), while others reported no differences (Raine and Venables, 1987) or even reduced CNV responses (Walter et al., 1964; McCalloum, 1973). Rockstroh et al. (1982) emphasizing cognitive rather than emotional aspects as main sources of the CNV. Therefore, the enhanced iCNV during CS+ compared to CS− trials in participants with pronounced interpersonal deficits reflected heightened attention or interest in the conditioned face stimuli, while the antisocial facet showed an inverse effect. With respect to the tCNV, the increased tCNV differentiation in subjects scoring high on the interpersonal facet might be interpreted in the context of preparedness, cognitive appraisal and contingency evaluation and can be considered as a further proof of superior cognitive processing without affecting emotional fear learning.

In line with previous studies, we found deficient fear conditioning in incarcerated, highly psychopathic offenders as indicated by diminished SCR differentiation between types of conditioned stimuli. Stepwise regression analysis revealed that only the affective facet is responsible for the low fear responses. Socialization is to a high degree based on the learning of stimulus-response (classical conditioning) and stimulus-reinforcement (instrumental conditioning) associations to adequately adapt behavior. A weak SCR differentiation during fear conditioning can therefore be an indication of maladaptive learning and failed socialization. Indicated by the event related potentials, we found a rather inferior early (N100) attentional but superior late (P300) attentional processing in subjects scoring high on the affective facet. Regarding the CNV responses, supposedly reflecting cognitive processing (Rockstroh et al., 1982), the interpersonal facet was associated with stronger CNV responses to the CS+ compared to the CS−, while the antisocial facet revealed the opposite effect. Such a “diaschisis” between the emotional and cognitive processing was often proposed in descriptive (Cleckley, 1941/1982), legal (Sommer et al., 2006) and artistic (Musil, 1930-1943) accounts and explanations of psychopathic criminals. This discrepancy between emotional and cognitive processing is not only mirrored by the reported discrepancy between the cognitive and emotional awareness of the aversive stimulus, but also obvious in empathy tasks with psychopathic individuals. Psychopaths demonstrate a complete failure to experience emotional empathy (Vollm et al., 2006) and this dysfunction was particularly evident in psychopaths with affective/interpersonal deficits (Decety et al., 2013). The psychopathic lack of emotional empathy seems to relate to disrupted affective processing and production. On the other hand, psychopaths are able to complete theory of mind tasks that require perspective taking without much difficulty (Richell et al., 2003).

## Limitations

Firstly, the generalizability of our results is limited by the small sample size and the lack of an adequate control group. By including more subjects, it would have been possible to validate the existent findings of differences between non-psychopathic and psychopathic subjects. In our study we attempted to capture psychopathic patients scoring high on PCL-R. A broader spectrum of psychopathy scores would be desirable to verify the dimensional relations we observed in the different subtypes of psychopathy.

We computed the Post hoc power analysis (two-tailed) for the significant bivariate correlations using the sample size, the effect size and the alpha error probability (0.05). Regarding the strongest correlation between PCL-R total scores and SCR, we calculated a power of 0.86 (one-tailed 0.92), and for the correlation with the affective facet a power of 0.70 (one-tailed 0.81). The power for the significant correlations between the EEG measures and psychopathy scores ranged from 0.58 to 0.88 (one-tailed 0.71–0.94). For that, we can assume that the actual power of the presented findings is moderate to large. Other critical points are the limited number of trials during the conditioning procedure. One reason for the relatively low number of trials was the fact that we used the classical conditioning design as a part of a comprehensive investigation conducted in the forensic institutions. Rothemund et al. (2012) used a quite similar design including the same face stimuli as CS and an electric shock as US. During the acquisition procedure they used 48 CS+ and 48 CS− trials, while in our study we presented 32 CS+ and 32 CS− trials. Rothemund and colleagues found remarkable differences between psychopathic participants and non-psychopathic control subjects in subjective, peripheral and electrocortical measures. In addition, Flor et al. (2002) and Birbaumer et al. (2005) showed in their conditioning experiments with psychopaths and non-psychopathic controls the same faces as CS as we used and both found successful conditioning in the relevant outcome measures in the control group. Therefore we can conclude that the fear conditioning deficit in terms of a reduced anticipatory SCR in psychopathic individuals, in particular with high scores on the affective facet, is specific to the group and not to the task.

The selection of the electrode placement already proofed to be sufficient in another task (Strehl et al., 2006) and refers to our specific questions concerning the ERP measurements, mainly focusing in the CNV changes in response to the CS (with both, iCNV and tCNV showing their maximal amplitude on FCz and Cz). Finally, the generalizability of our results is also limited by the fact that till now no study exists, investigating fear conditioning in female, psychopathic inmates. A corresponding study would help to understand the underlying mechanism of this psychopathy-related physiological manifestation.

## Conclusion

In conclusion the diminished peripheral-emotional response (SCR) to aversive events in our study seems to be attended by inferior sensory and superior cognitive processing in more affective/interpersonal deficient psychopaths. Therefore, especially the aberrant cognitive-emotional interaction in psychopathy seems to be the key in fear conditioning as indicated by the subjective, peripheral-physiological and electrophysiological data. The present findings hint at segregated emotional and cognitive processing during implicit fear learning in psychopathic subtypes. This is of special importance and could have profound implications for the research on psychopathy including externalizing psychopathology. Without doubt, more studies are needed to shed light on the different cortical as well as peripher-physiological processes associated with the subtypes, facets and related shortcomings of psychopathy.

### Conflict of interest statement

The authors declare that the research was conducted in the absence of any commercial or financial relationships that could be construed as a potential conflict of interest.
